# The universal tree of life: an update

**DOI:** 10.3389/fmicb.2015.00717

**Published:** 2015-07-21

**Authors:** Patrick Forterre

**Affiliations:** ^1^Unité de Biologie Moléculaire du Gène chez les Extrêmophiles, Département de Microbiologie, Institut Pasteur, Paris, France; ^2^Institut de Biologie Intégrative de la cellule, Université Paris-Saclay, Paris, France

**Keywords:** archaea, bacteria, eukarya, LUCA, universal tree, evolution

## Abstract

Biologists used to draw schematic “universal” trees of life as metaphors illustrating the history of life. It is indeed a priori possible to construct an organismal tree connecting the three major domains of ribosome encoding organisms: Archaea, Bacteria and Eukarya, since they originated by cell division from LUCA. Several universal trees based on ribosomal RNA sequence comparisons proposed at the end of the last century are still widely used, although some of their main features have been challenged by subsequent analyses. Several authors have proposed to replace the traditional universal tree with a ring of life, whereas others have proposed more recently to include viruses as new domains. These proposals are misleading, suggesting that endosymbiosis can modify the shape of a tree or that viruses originated from the last universal common ancestor (LUCA). I propose here an updated version of Woese’s universal tree that includes several rootings for each domain and internal branching within domains that are supported by recent phylogenomic analyses of domain specific proteins. The tree is rooted between Bacteria and Arkarya, a new name proposed for the clade grouping Archaea and Eukarya. A consensus version, in which each of the three domains is unrooted, and a version in which eukaryotes emerged within archaea are also presented. This last scenario assumes the transformation of a modern domain into another, a controversial evolutionary pathway. Viruses are not indicated in these trees but are intrinsically present because they infect the tree from its roots to its leaves. Finally, I present a detailed tree of the domain Archaea, proposing the sub-phylum neo-Euryarchaeota for the monophyletic group of euryarchaeota containing DNA gyrase. These trees, that will be easily updated as new data become available, could be useful to discuss controversial scenarios regarding early life evolution.

## Introduction

The editors of research topic on “*archaeal cell envelopes and surface structures*” gave me the challenging task of drawing an updated version of the universal tree of life. This is a daunting task indeed, given that the concept of a universal “tree” is disputed by some scientists, who have suggested replacing trees with networks, and that major features of the tree are still controversial (Gribaldo et al., 2010; Forterre, 2012). Thus, it will be difficult to draw a consensus tree welcomed by all scientists in the field. In this paper, I thus try to propose updated versions of the universal tree that include as many features as possible validated by robust phylogenetic analyses and/or comparative molecular biology and biochemistry. I will draw a “universal tree” limited to ribosome-encoding organisms (Raoult and Forterre, 2008) that diverged from the last universal common ancestor (LUCA). Viruses (capsid encoding organisms) are polyphyletic, therefore their evolution can be neither illustrated by a single tree nor included in the universal tree as additional domains (Forterre et al., 2014a). However, this should not be viewed as neglecting the role of viruses in biological evolution because “*the tree of life is infected by viruses from the root to the leaves*” (Forterre et al., 2014a). The universal tree of ribosome-encoding organisms contains cellular organisms that, unlike viruses, reproduce via cell cycles that imply the formation of new cells from the division of mother cells. This implies a fascinating continuity in the heredity of the cell membrane from LUCA to modern members of the three domains. A robust universal tree is critical to make sense of the evolution of the several types of lipids, cell envelopes and surface structures that originated and evolved on top of this continuity.

## The Textbook Trees

Several popular drawings of the universal tree of life are widespread in the scientific literature and textbooks. These include the radial rRNA tree of Pace (1997), the tree of Stetter (1996) rooted in a hyperthermophilic LUCA and the most famous tree, which was published by Woese et al. (1990) to support their proposal to change the name “Archaebacteria” to Archaea and to define the Domain as the highest taxonomic level (Woese et al., 1990; Sapp, 2009). All these trees are based on ribosomal DNA (rDNA) sequence comparisons and are rooted between Bacteria and a common ancestor of Archaea and Eukarya, a rooting that was first suggested by phylogenetic analyses of paralogous proteins (Iwabe et al., 1989; Gogarten et al., 1989).

The rDNA trees are still widely used despite their age (from 17 to 25 years old) because scientists need metaphors to represent the history of living organisms (despite all criticisms to the tree concept itself) and because few new trees have been proposed in the past two decades that are accepted by most biologists. Doolittle (2000) published a widely popularized tree in which the bases of the three domains are mixed by widespread lateral gene transfer (LGT). However, studies on archaeal phylogeny and more recently in Bacteria and Eukarya, as well as comparative genomic analyses, have shown that the history of organisms (not to be confused with the history of genes) can be inferred from a core of highly conserved genes (Brochier et al., 2005a; Gribaldo and Brochier, 2009; Puigbò et al., 2013; He et al., 2014; Ramulu et al., 2014; Raymann et al., 2014; Petitjean et al., 2015).

Comparative genomics has confirmed the existence of three versions (*sensu* Woese) of all universally conserved proteins, validating the three domains concept at the genomic level and opening the way to protein based universal trees (Olsen and Woese, 1997). A fairly popular radial tree based on a set of universal proteins was published in 2006 by Bork and colleagues (Ciccarelli et al., 2006). Unfortunately, this tree is biased by the over-representation of bacteria, because of the method used to create it, which requires complete genome sequences. Furthermore, several detailed internal branches within each domain are either controversial, such as the presence of Chlamydiae and Planctomycetes in different bacterial superphyla (Kamke et al., 2014). A well-thought-out universal tree based on rRNA was published in by López-García and Moreira (2008). This unrooted tree depicts each domain as a radial form with many phyla, without resolving the relationships between phyla within domains. However, like all previous trees, this tree does not show some major lineages identified in the past decade, such as the Thaumarchaeota, which are now recognized as one of the three major archaeal phyla (Brochier-Armanet et al., 2008a; Spang et al., 2010, 2015).

## Problems with Textbook Trees

The universal trees of the 1990s based on rDNA that are still widely used in textbooks, reviews and seminars provide a misleading view of the history of organisms. For instance, they all depict the division of Eukarya between a crown including Plants, Metazoa, Fungi, and several lineages of protists, and several basal long branches leading to various other unicellular eukaryotes, of which the most basal are protists lacking mitochondria (formerly called Archaezoa). This topology of the eukaryotic tree was very popular in the 1990s but is the result of a long branch attraction artifact. At the beginning of this century, it was acknowledged that all major eukaryotic divisions should be somewhere in the crown (Embley and Hirt, 1998; Keeling and McFadden, 1998; Philippe and Adoutte, 1998; Gribaldo and Philippe, 2002).

Another problem still present in many textbook trees is the position of hyperthermophiles. All rDNA trees of the 1990s were rooted within hyperthermophilic archaea and bacteria (Woese et al., 1990; Stetter, 1996; Pace, 1997). In particular, the hyperthermophilic bacteria of the genera *Thermotoga* and *Aquifex* were the two most basal bacterial lineages in all these trees. This explains why these bacteria are still often labeled as “deep-branching bacteria” (Braakman and Smith, 2014). However, the analysis of ribosomal RNA sequences at slowly evolving nucleotide positions (Brochier and Philippe, 2002) and phylogenetic analyses based on protein sequences do not support the deep branching of *Thermotoga* and *Aquifex* in the bacterial tree (Boussau et al., 2008b; Zhaxybayeva et al., 2009). The exact position of these hyperthermophilic bacteria currently remains controversial, because of the unusual extent of LGT that occurred between these bacteria and some other bacterial groups (Boussau et al., 2008b; Zhaxybayeva et al., 2009; Eveleigh et al., 2013).

The clustering of hyperthermophiles around the root and their “short branches” have been widely cited as support for the idea of a hot LUCA and a hot origin of life (Stetter, 1996), although the phenotype of an organism at the tip of a branch does not necessarily reflect that of its ancestor at the base. However, early reports noted that both features could be explained by the very high GC content of the ribosomal RNA of hyperthermophiles that limits the sequence space available for the evolution of these molecules (Forterre, 1996; Galtier et al., 1999; Boussau et al., 2008a). Indeed, the reconstruction of ribosomal RNA and protein sequences in LUCA shed serious doubt on its hyperthermophilic nature, and suggests instead that it was either a mesophilic or a moderate thermophilic organism (Galtier et al., 1999; Boussau et al., 2008a). This result is in agreement with the facts that specific thermoadaptation features of lipids in Archaea and Bacteria are not homologous and that reverse gyrase, a protein required for life at very high temperature, was probably not present in the common ancestor of Archaea and Bacteria (Forterre, 1996; Brochier-Armanet and Forterre, 2007; Glansdorff et al., 2008). These observations suggest that thermal adaptation from LUCA to the ancestors of Archaea and Bacteria took place from cold to hot and not the other way around.

## A Tree or a Ring?

Several authors during the past three decades have proposed to replace the universal tree with a “ring of life” (*sensu* Rivera and Lake, 2004) because they think that Eukarya originated from the association of a bacterium with an archaeon (for recent reviews, see Forterre, 2011; Martijn and Ettema, 2013). The most recent version of ring of life scenario is that eukaryogenesis was triggered by the engulfment of an alpha-proteobacterium by a wall-less giant archaeon capable of phagocytosis (Martijn and Ettema, 2013). Proponents of fusion (association) scenarios argue that such fusion is required to explain why eukaryotic genomes contain both archaeal and bacterial-like genes. However, this is not the case, since the presence of archaeal-like genes in Eukarya is a logical consequence of the sisterhood of Archaea and Eukarya, whereas the presence of bacterial-like genes is the expected result of mitochondrial endosymbiosis. Additional bacterial genes might have been introduced in proto-eukaryotes by LGT (Doolittle, 1998), which may have been partly mediated by large DNA viruses (Forterre, 2013a). Finally, ring of life scenarios do not easily explain the presence of many core eukaryotic genes (around 40%) that were already present in the last eukaryotic common ancestor (LECA) but have no detectable bacterial or archaeal homologs (Fritz-Laylin et al., 2010).

Ring of life scenarios, as well as scenarios in which Eukarya emerged from within Archaea (see below) assume the transformation of one and/or two of the modern domain into a third one. These scenarios have been criticized by several authors, as being biologically unsound (Woese, 2000; Kurland et al., 2006; De Duve, 2007; Cavalier-Smith, 2010; Forterre, 2011, 2013a). In particular, Woese (2000) argued that: “*modern cells are sufficiently integrated and “individualized” that further change in their designs does not appear possible.*” However, even if eukaryogenesis was actually triggered by the endosymbiotic event that produced mitochondria, this would not be a good reason to replace the tree of life with a ring. The universal tree should depict evolutionary relationships between domains defined according to the translation apparatus reflecting the history of cells (and their envelope; Woese et al., 1990) and not according to the global genomic composition that is influenced by LGT, virus integration and endosymbiosis, the history of which is incredibly complex.

**FIGURE 1:**
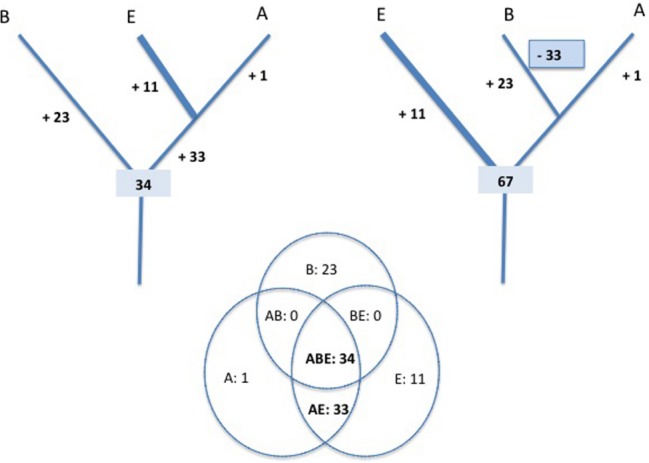
**Evolution of the ribosome proteins content.** The universal tree is rooted in the bacterial branch (left) or in the eukaryotic branch (right). In each case, the most parsimonious scenarios for the evolution of ribosomal proteins content are presented. The numbers of proteins present at each evolutionary steps are deduced from the distribution of homologous ribosomal proteins in the three domains of life, Archaea (A), Bacteria (B), and Eukarya (E) (adapted from the data of Lecompte et al., 2002).

This is well illustrated by the case of Plantae. The endosymbiosis of a *Cyanobacterium* that created this eukaryotic megagroup don’t prevent evolutionists to draw a tree of Eukarya in which Plantae are represented as one branch of the tree, and not as the product of a ring (He et al., 2014). The tree of any particular taxonomic unit is indeed not affected by the presence (or absence) of endosymbionts in some of its branches! Thus, a universal tree of life depicting the three domains as three separate entities does not contradict the fusion/endosymbiotic hypotheses at the origin of Eukarya, as long as this event had no effect on the eukaryotic ribosome itself. This is not the case, because the eukaryotic ribosome is not a mixture of archaeal and bacterial ribosomes; it shares 33 proteins with archaeal ribosomes that are not present in Bacteria, but none with the bacterial ribosome that are not present in Archaea (Lecompte et al., 2002; Figure 1).

## The “eocyte” Question

In the 1980s, James Lake proposed a universal tree in which Eukarya are sister group of a subgroup of Archaea (later recognized as Crenarchaeota) that he called Eocytes (Lake et al., 1984; Sapp, 2009). Most phylogeneticists now support a new version of the “Eocyte tree,” in which Eukarya emerge from within Archaea, and are a sister group of a superphylum encompassing Thaumarchaeota, Aigarchaeota, Crenarchaeota, and Korarchaeota (the TACK superphylum; Guy and Ettema, 2011; Williams et al., 2013; McInerney et al., 2014; Raymann et al., 2015), a sister group of Thaumarchaeota (Katz and Grant, 2014) or a sister group of Korarchaeota (Raymann et al., 2015). In a recent study, Eukarya are even proposed to have emerge from within a new phylum of the TACK superphylum (tentative phylum Lokiarchaeota; Spang et al., 2015).

The “eocyte” scenario is supported by phylogenetic analyses of universal proteins that use sophisticated methods for tree reconstruction, which are thought to be very efficient at identifying weak phylogenetic signals. However, these data are controversial, because most universal proteins are small (e.g., ribosomal proteins) and very divergent between Bacteria and Archaea/Eukarya, which makes archaeal/eukaryal relationships difficult to resolve (Gribaldo et al., 2010). For instance, the elongation factor datasets are saturated and unable to identify deep phylogenetic relationships between eukaryal phyla (Philippe and Forterre, 1999), and it is therefore challenging to use them as phylogenetic markers to resolve even deeper evolutionary relationships. In fact, in the single trees obtained for universal proteins by Cox et al. (2008), Eukarya branch within Euryarchaeota in about half of the trees and within Crenarchaeota in the other half, and they are characterized by poor node resolution and many aberrant groupings within Archaea (Cox et al., 2008, supporting information online). Similar unresolved and contradictory single-gene trees were again obtained by Williams and Embley in their more recent universal phylogeny (see supplementary Figure S1 in Williams and Embley, 2014).

One should be cautious in the interpretation of trees obtained from the concatenation of protein sequences that produce such contradictory individual trees. Indeed, the Microsporidia *Encephalitozoon*, a derived fungus, appears at the base of the eukaryotic tree published by Cox et al. (2008). This is reminiscent of the phylogenies of the 1990s that misplaced Microsporidia and other amitochondriate eukaryotes at the base of the tree of Eukarya (Hashimoto et al., 1995; Kamaishi et al., 1996). Similarly, several long basal eukaryotic branches (Fornicata, Archamoeba) emerged between Thaumarchaeota and other Eukarya in the tree of Katz and Grant (2014). In the tree supporting the emergence of Eukaryotes from Lokiarchaeota, the archaeal tree is rooted in the branch leading to *Methanopyrus kandleri* (Spang et al., 2015) a fast evolving archaeon (Brochier et al., 2004). Finally, in the analysis of Gribaldo and coworkers, the archaeal tree is rooted between Euryarchaeota and the putative TACK superphylum using eukaryotic proteins as outgroup, but it is rooted in the branch leading to Korarchaeota when universal bacterial proteins are added to the dataset (Raymann et al., 2015).

The universal tree published by Gribaldo and co-workers reflects our best present knowledge of the internal branching order within Archaea and recovers the monophyly of most phyla in the three domains (Raymann et al., 2015). Notably, Archaea are rooted in this tree within Euryarchaeota when bacterial proteins are used as an outgroup, suggesting a new root for Archaea. However, Moreira and colleagues found instead that the root of the archaeal tree is located between Euryarchaeota and Crenarchaeota when using a bacterial outgroup (Petitjean et al., 2014).

**FIGURE 2:**
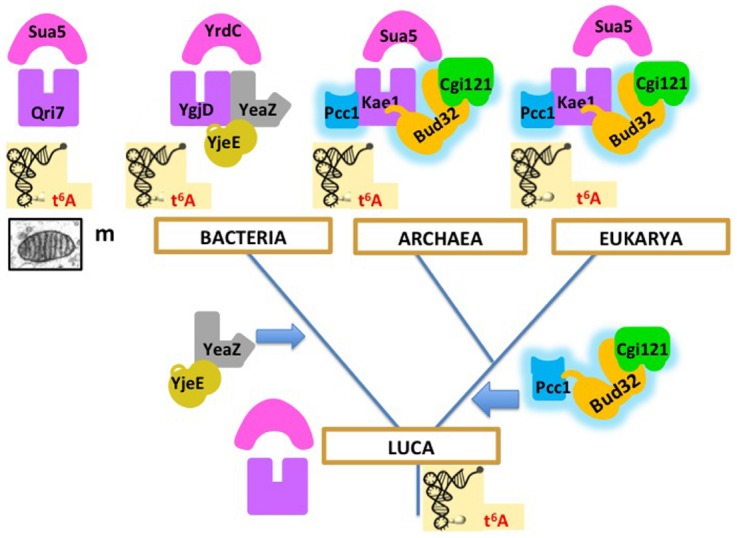
**Evolution of the biosynthesis pathway of threonylcarbamoyl adenosine (t6A) from LUCA to the three domains of life.** Homologous proteins have the same shapes and colors, except for YgjE and YeaZ, which are homologous but not orthologous. In this scenario, this universal tRNA modification was performed in LUCA by two universal proteins (the ancestors of Kae1/YgjD and of Sua5/YrdC, respectively), as in mitochondria (Wan et al., 2013; Thiaville et al., 2014). Additional proteins are now essential for t6A synthesis in modern organisms (Deutsch et al., 2012; Perrochia et al., 2013a,b). The bacterial proteins (YjeE, YeaZ) are non-homologous to the archaeal/eukaryal ones (Bud32, Pcc1, Cgi121). The most parsimonious scenario supports the rooting between Bacteria and Archaea/Eukarya, because other roots would require the presence of the Bud32, Pcc1, and Cgi121 proteins in LUCA, and their replacement by the YeaZ, YjeE proteins in Bacteria, an unlikely evolutionary pathway (see text).

I previously reported an observation that questions the validity of the methods used for tree reconstruction in some of these analyses. The three domains are each monophyletic, with well resolved evolutionary relationships within domains, in a tree of the universal protein Kae1/YgjD published in 2007 (see Figure S1 in Hecker et al., 2007). In contrast, Archaea are paraphyletic for the same protein in the analysis of Cox et al. (2008) see Figure 2 in Forterre, 2013a) or in a more recent analysis by the same research group (Williams and Embley, 2014, supporting information online). In the tree of Cox et al. (2008), Eukarya are a sister group of a clade containing crenarchaea and the euryarchaeon *Methanopyrus kandleri*, whereas in the tree of Williams and Embley, eukaryotic Kae1 emerges from a clade grouping Methanobacteriales and Methanoccoccales. This illustrates the importance to present in supplementary material the individual trees of universal proteins beside those obtained with concatenation of protein sequences.

The various sets of universal proteins used by different groups to investigate the relationships between Archaea and Eukarya show substantial overlap and it is probable that most protein data sets lack valid phylogenetic signal (Gribaldo et al., 2010). Two groups that analyzed similar sets of proteins with various methods came to a similar conclusion (Lasek-Nesselquist and Gogarten, 2013; Rochette et al., 2014). Lasek-Nesselquist and Gogarten (2013) noticed that “*the methods used*” to recover the eocyte tree “*generate trees with known defects,…revealing that it is still error prone*,” whereas Rochette et al. (2014) concluded that “*the high frequency of paraphyletic-Archaea topologies for near-universal genes may be the consequence of stochastic effects*.”

Generally speaking, it is very difficult to resolve ancient relationships by molecular phylogenetic methods for both practical and theoretical reasons, essentially because the informative signal is completely erased at long evolutionary distances (Forterre and Philippe, 1999; Mossel, 2003; Penny et al., 2014). One possibility to bypass this phylogenetic impasse is to focus on biological plausibility. Trees in which the three domains are each monophyletic are more plausible than trees in which Archaea are monophyletic because they explain more easily the existence of three versions of the ribosome discovered by Woese et al. (1990). In the classical Woese tree, these versions emerged from ancestors that differed from modern cells, at a time when the tempo of protein evolution was faster than today. By contrast, scenarios in which Eukarya emerged from within Archaea assume that members from a modern domain (Archaea) were transformed recently (after the diversification of this domain) into completely different organisms (Eukarya), something difficult to imagine from a biological point of view (Woese, 2000; Kurland et al., 2006; De Duve, 2007; Cavalier-Smith, 2010; Forterre, 2011, 2013a).

It has been proposed that this dramatic transformation (implying among others the replacement of archaeal type lipids by bacterial type lipids) was initiated in a particular archaeal lineage that, in contrast to all other lineages, already evolved toward more complex forms before eukaryogenesis (Martijn and Ettema, 2013). The recently proposed novel archaeal phylum, Lokiarchaeota, appears to be a good candidate in that case because its genome apparently encodes many genes potentially involved in the manipulation of membranes or in the formation of a cytoskeleton, including several homologs of eukaryal proteins that are observed for the first time in Archaea (Spang et al., 2015). Eukaryotic-like features present in Archaea (the archaeal eukaryome, *sensu* Koonin and Yutin, 2014) are indeed widely dispersed among the various archaeal phyla (Koonin and Yutin, 2014), suggesting that all these features were present in the last archaeal common ancestor (LACA), which was more complex than modern archaea (Forterre, 2013a). This observation is easily explained in the framework of the Woese tree by the selective loss of these features (present in the last common ancestor of Archaea and Eukarya) in different archaeal phyla during the streamlining process that led to the emergence of modern archaea (Forterre, 2013a). Comparative genomic analyses have indeed previously revealed a tendency toward reduction in the evolution of Archaea (Csurös and Miklos, 2009; Makarova et al., 2010; Koonin and Yutin, 2014).

In contrast, in the eocyte scenario, most eukaryotic features present in Archaea originated during the transition between Euryarchaeota and the TACK superphylum. This scenario further implies that all archaea “stopped evolving,” remaining archaea, whilst progressively and randomly losing some of these eukaryotic features, except for one particular lineage of Lokiarchaeota that experienced a dramatic burst of accelerated evolution and was transformed into eukaryotes.

It is traditionally suggested that the process that led to this transformation was triggered by the endosymbiosis event that created mitochondria (Lane and Martin, 2010). This seems to be a leap of faith, because there is no example of such a drastic transformation of the host molecular fabric at the more basic and fundamental levels (translation, transcription, replication) triggered by an endosymbiotic event (Forterre, 2013a). For instance, Plantae remain *bona fide* Eukarya (with typical eukaryotic version of all universal proteins) despite the fact that about 20% of their genes originated from cyanobacteria (Martin et al., 2002).

An argument often used for scenarios in which Eukarya descended from modern lineages of prokaryotes is that Eukarya only appeared recently in the fossil record (McInerney et al., 2014). Most authors supporting this scenario systematically ignore the discovery 5 years ago of possible multicellular eukaryotes in sediments dating 2.1 billions years old (El Albani et al., 2010). This suggests that the last common ancestor of modern eukaryotes could have originated much earlier than previously thought (well before 2.1 Gyr ago) and that proto-eukaryotes could have been already present even earlier. In any case, this also confirms that the tempo of evolution of the central components of the molecular cell fabric has decreased around three Gyr ago, possibly after the emergence of the three domains, as first suggested by (Woese, 2000; Forterre, 2006). This also explains why it is so challenging to determine the precise topology of the universal tree of life, considering that we are dealing with six Gyr of evolution, encompassing periods with very different evolutionary tempo, when we are comparing two modern sequences of universal proteins.

## The Elusive Root of the Tree

A major problem in drawing the universal tree of life is the position of the root. The tree is rooted between Bacteria and Archaea/Eukarya in the classical Woese tree (Woese et al., 1990). This rooting was initially supported by phylogenetic analyses of protein paralogs (elongation factors and V/F types ATPase subunits) that originated by duplication before LUCA (Gogarten et al., 1989; Iwabe et al., 1989). This rooting was criticized in the 1990s because the bacterial branches are much longer than the other two branches in the trees of these protein paralogs (Forterre and Philippe, 1999; Philippe and Forterre, 1999). Furthermore, the elongation factors and V/F type ATPase subunits data sets, as well as other groups of paralogs (e.g., signal recognition particles, SRP) that also used to place the root between Bacteria and a common ancestor of Archaea and Eukarya were saturated with mutations (Philippe and Forterre, 1999). Therefore, it was unclear if this rooting reflects the real history of life on our planet or if it is due to a long branch attraction artifact, e.g., the “bacterial branch” being attracted by the long branch of the outgroup sequences of the paralogs. Statistical analysis of slowly evolving positions in the two paralogous subunits of SRP confirmed that the bacterial rooting obtained by more classical phylogenetic analyses with SRP was due to a long branch attraction artifact and suggested that the root is located between Archaea/Bacteria and Eukarya (Brinkmann and Philippe, 1999); however, this analysis was not followed up.

As is the case for archaeal/eukaryal relationships, there is probably no valid phylogenetic signal left in the universal protein data set to resolve the rooting of the universal tree by molecular phylogeny. This was confirmed in the case of the elongation factors data set by a cladistic analysis of individual amino-acid alignments that discriminate between primitive and share derived characters (Forterre et al., 1992). Only 23 positions could be subjected to this analysis in the elongation factor data set, of which 22 gave ambiguous results and only one supported bacterial rooting!

These past 20 years, the rooting problem has been neglected—with a few exceptions (see for instance Harish et al., 2013) that I have no space to discuss here. Indeed, “ring of life” scenarios or those in which Eukaryotes originated from Archaea automatically root the tree between Archaea and Bacteria (rejuvenating the pre-Woesien prokaryote/eukaryote paradigm). However, comparative molecular biology has now revealed several situations that can help us to root the universal tree and decide between alternative scenarios.

## Rooting From Comparative Molecular Biology

Comparative genomic analyses have shown that most proteins central for cellular function (both informational and operational) show higher sequence similarity between Archaea and Eukarya than between Eukarya and Bacteria. Furthermore, Archaea and Eukarya also share many proteins that are either absent in Bacteria or replaced with non-homologous proteins with the same function. Surprisingly, comparative genomic analyses have also shown that critical components of the DNA replication machinery (replicase, primase, helicase) are non-homologous between Archaea/Eukarya and Bacteria (Leipe et al., 1999; Forterre, 2006). This is also the case for the proteins that allow the bacterial F-type ATPase and archaeal A-type ATPase to work as ATP synthases (Mulkidjanian et al., 2007). All these observations are difficult to interpret if the universal tree is rooted in the archaeal or eukaryotic branches and/or if the archaeal/eukaryal specific proteins were present in LUCA. Indeed, this would have required many non-orthologous replacement events that occurred specifically in the bacterial branch. Figure 1 illustrates the case of the ribosome evolution. The eukaryotic (or archaeal) rooting is clearly less parsimonious than the bacterial ones since it implies the loss of 33 ancestral ribosomal proteins in the bacterial branch concomitant with the gain of 23 new proteins. Such non-orthologous replacement scenario cannot be completely ruled out since a similar gain and loss event occurred during the evolution of the mitochondrial ribosome from the bacterial one (Desmond et al., 2011). However, there is no evidence that the emergence of bacteria involved a dramatic evolutionary event similar to the drastic reductive evolution that occurred during the emergence of mitochondria.

It was previously suggested that non-orthologous replacement had indeed occurred for the DNA replication machinery, with the ancestral DNA replication machinery in LUCA being replaced by non-homologous DNA replication proteins of viral origin either in Bacteria or in Archaea/Eukarya (Forterre, 1999). However, this type of explanation cannot be easily generalized. It seems unlikely that multiple non-orthologous replacements can explain all other major differences between archaeal/eukaryal and bacterial analogous but non-homologous systems! In the case of the DNA replication machineries, it is simpler to imagine that two versions present in modern cells were independently transferred from viruses to cells, once in the bacterial lineage and once in the archaeal/eukaryal lineage (Mushegian and Koonin, 1996; Forterre, 2002, 2013b). For instance, our preliminary analyses of universal proteins sequence alignments indicate that the Lokiarchaeon is probably neither an early branching archaeon nor a missing link between Archaea and Eukarya (see also Nasir et al., 2015). Similarly, other analogous, but non-homologous, systems, such as the two distinct rotary proteins involved in ATP synthesis by F°/F1 and A/V ATPases, might have originated independently in the bacterial and in the archaeal/eukaryal lineages (Mulkidjanian et al., 2007).

Another explanation for the existence of non-homologous systems between Archaeal/Eukaryal and Bacteria is that LUCA contained two redundant systems and that one of them was later on lost at random in each domain (Edgell and Doolittle, 1997; Glansdorff et al., 2008). However, it is unlikely that both versions of all non-homologous systems between Archaeal/Eukaryal and Bacteria were present in LUCA. For instance, no modern cells have two non-homologous versions of DNA replication machineries or two versions of RNA polymerases (the bacterial and the archaeal ones). Some systems could have been randomly distributed between LUCA and other contemporary cellular (or viral) lineages, and redistributed thereafter by LGT, but this seems very unlikely in the case of the ribosome.

Recent biochemical work in our laboratory exemplifies why comparative biochemistry data support a universal tree in Archaea and Eukarya are indeed sister domains. Several research groups, as well as our team in Orsay, succeeded in reconstituting *in vitro* the protein complexes involved in the biosynthesis of the universal threonylcarbamoyl adenosine (t6A) tRNA modification in position 37 of tRNA in the three domains of life (Deutsch et al., 2012; Perrochia et al., 2013a,b) and in mitochondria (Wan et al., 2013; Thiaville et al., 2014). In Bacteria, Archaea and Eukarya, the reactions require the combination of two universal proteins and essential accessory proteins that exist in two versions, one present in Bacteria, the other present in Archaea and Eukarya. Interestingly, the same reaction can be performed in mitochondria by the two universal proteins alone, one (Qri7) that came from Bacteria via the endosymbiotic route and the other (Kae1) corresponding to the eukaryotic version (Wan et al., 2013; Thiaville et al., 2014). These results suggest that LUCA was able to perform this universally conserved reaction with the ancestors of the two universal proteins and that accessory proteins (now essential) were added independently in the bacterial and in the archaeal/eukaryal lineages. The most parsimonious scenario, illustrated in Figure 2, supports the rooting between Bacteria and Archaea/Eukarya, because other roots would require the presence of the archaeal/eukaryal set of accessory proteins in LUCA, and its replacement by the non-homologous bacterial set in Bacteria. This seems unlikely because biochemical analyses have shown that the bacterial and archael/eukaryal accessory proteins are not functionally equivalent (Deutsch et al., 2012; Perrochia et al., 2013b). It is therefore difficult to imagine intermediate steps in the replacement process. Furthermore, such replacement, even partial, never occurred during the diversification of the three domains.

Woese and Fox (1977) were thus possibly right when they proposed that the molecular fabric of LUCA was simpler than that of modern organisms, and that this organism still had an RNA genome. In this scenario, major molecular machineries, such as the DNA replication machineries or the ATP synthases, emerged and/or became sophisticated independently in the branches leading to Bacteria on one side and to the common ancestor of Archaea and Eukarya on the other.

**FIGURE 3:**
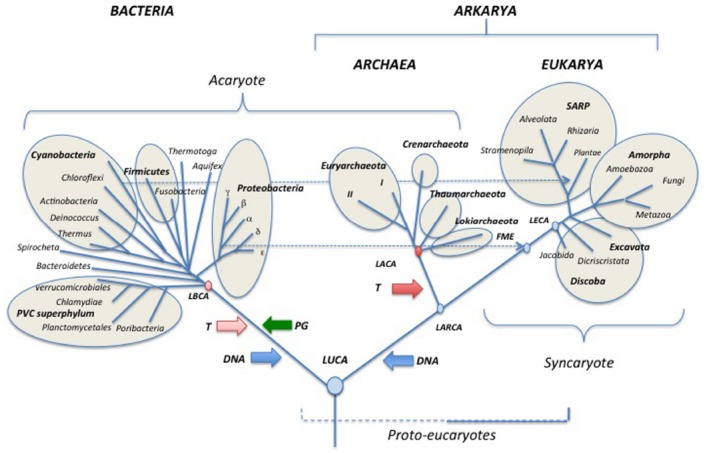
**Schematic universal tree updated from (Woese et al., 1990; see text for explanations).** PG: peptidoglycan; DNA (blue arrows) introduction of DNA; T (pink and red arrows) thermoreduction. LBCA: Last Bacterial Common Ancestor, pink circle: thermophilic LBCA; LACA: Last Archaeal Common Ancestor, red circle, hyperthermophilic LACA. LARCA: Last Arkarya Common Ancestor; FME: First Mitochondriate Eukarya; LECA: Last Eukaryotic Common Ancestor; blue circles, mesophilic ancestors. SARP: Stramenopila, Alveolata, Rhizobia, Plantae.

**FIGURE 4:**
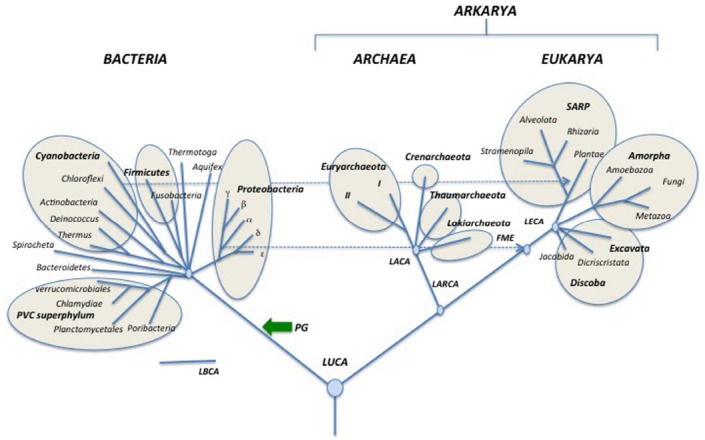
**Schematic simplified universal tree updated from Woese et al. (1990). Abbreviations are the same as in Figure 3**.

**FIGURE 5:**
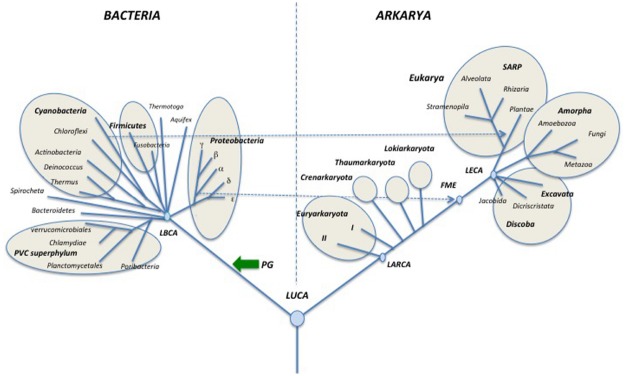
**Schematic universal tree updated from Woese et al. (1990) and modified according to the “eocyte” hypothesis.** Abbreviations are the same as in Figure 3. In this configuration, Archaea is no more a valid taxon since “Archaea” are paraphyletic (LACA is also an ancestor of Eukarya) suggesting using the suffix karyota for the various “archaeal” phyla. Together with Eukarya, these phyla became various phyla of Arkarya.

The rooting of the universal tree in the so-called “bacterial branch” (Figures 3–5) has been often interpreted as suggesting a “prokaryotic phenotype” for LUCA. This is a misleading interpretation that again confuses the phenotypes at the tip and base of a branch. The rooting between a lineage leading to Bacteria and a lineage leading to Archaea and Eukarya is compatible with diverse types of LUCA, including a LUCA with some “eukaryotic-like features” that were lost in Archaea and Bacteria (Forterre, 2013a).

Importantly, rooting of the universal tree in the “bacterial branch” formally requires giving a name to the clade grouping Archaea and Eukarya. Woese (2000) never proposed such a name, adopting a “gradist” view of life evolution, with the three Domains emerging independently from a “communal LUCA” before the “Darwinian threshold” (Woese, 2000, 2002). In such view, the notion of clade itself cannot be used to group organisms that diverged at the time of LUCA when no real speciation occurred. I have criticized the Darwinian threshold concept, assuming—with many others—that Darwinian evolution started as soon as biological evolution take off (see Forterre, 2012, and references therein). In particular, extensive genes exchanges that possibly take place at the time of LUCA (but see Poole, 2009) cannot be opposed to Darwinian evolution occurring through variation and selection, since gene transfer only corresponds to a specific type of variation (Forterre, 2012).

I think that it’s time now to look back at the universal tree with a cladistics perspective and to propose a name for the clade grouping Archaea and Eukarya. It is challenging to find a common synapomorphy to Archaea and Eukarya that could provide a good name for the clade corresponding to these two domains. David Prangishvili suggests Arkarya (personal communication), combining the names of the two domains belonging to this clade (Figure 3). Notably, universal trees in which Eukarya emerge from within Archaea are often viewed as “two domains trees” *versus* the “three domains tree” of Carl Woese (Gribaldo et al., 2010). However, the new nomenclature proposed here emphasizes that the classical Woese tree is also *stricto sensu*, a two domains tree (Bacteria and Arkarya)!

## Updated Trees for Everybody

The backbone of the updated universal trees proposed here (Figures 3–5) was selected from the 1990 tree of Woese et al. (1990) as a tribute to Carl Woese and the historical work of the Urbana school (Sapp, 2009; Albers et al., 2013). The relative lengths of the branches linking the three domains together combine features of the rDNA and protein trees. It is indeed puzzling that Archaea and Eukaryotes are very close in trees based on universal protein sequence comparison (Rochette et al., 2014), but are more divergent in those based on rDNA (Pace et al., 1986). The reason for this discrepancy remains unclear and should be worth exploring further. The rather long branches between domains in the trees of Figures 3–5 also reflect the “three major transformation events” (*sensu* Forterre and Philippe, 1999) that occurred between LUCA and the formation of each domain (Woese, 1998; Forterre and Philippe, 1999).

Evidently, it is not possible to draw a tree including all presently recognized phyla, especially in the bacterial domain, so I made arbitrary choices, and tried to include most well studied bacterial and archaeal phyla, as well as major eukaryotic divisions and/or supergroups. In the case of Archaea, I only indicate the phyla Euryarchaeota, Crenarchaeota, Thaumarchaeota and the candidate phylum “Lokiarchaeota” because other proposed archaeal phyla are represented by a single species and/or their phylum status is controversial or has been refuted by robust phylogenetic and phylogenomic analyses (see below). Although still preliminary, the study of three partial lokiarchaeal genomes has shown that these archaea encode many eukaryotic-like genes absent in Thaumarchaea and are clearly separated from both Thaumarchaeota and Crenarchaeota in phylogenetic analysis (Spang et al., 2015). Furthermore, Lokiarchaeota correspond to a large clade of abundant and diversified uncultivated archaea, previously named deep-sea archaeal group (DSAG), that are widely distributed in both marine and fresh water environments (Jørgensen et al., 2013).

I divide the phylum Euryarchaeota in sub-phylum I (I) and sub-phylum II (II), according to the presence/absence of DNA gyrase (see below; Forterre et al., 2014b). Dotted lines indicate the endosymbiosis events that had a major impact on the history of life by triggering the emergence of both modern eukaryotes (mitochondria) and Plantae (chloroplasts). In particular, this reminds us that the first mitochondriate eukaryote (FME) emerged after the diversification of alpha proteobacteria, indicating that “modern eukarya” are indeed much more recent than Archaea and Bacteria.

In the tree of Figure 3, I use the terms “synkaryote” and “akaryote” (with and without a nucleus, respectively) instead of eukaryotes and prokaryotes (Forterre, 1992; Harish et al., 2013; Penny et al., 2014). This is because the latter terms are the hallmark of the traditional (pre-Woesian) view of the evolution of life from primitive bacteria (“pro” karyotypes) to lower and finally higher eukaryotes (Forterre, 1992; Pace, 2006; Penny et al., 2014).

Some major events that shaped modern domains are indicated, such as the introduction of peptidoglycan (PG) in the lineage leading to Bacteria. The last bacterial and archaeal common ancestors (LBCA and LACA) are colored in pink and red, respectively, to indicate their probable thermophilic and hyperthermophilic nature based on ancestral protein and rRNA sequence reconstruction (Boussau et al., 2008a; Groussin and Gouy, 2011; Groussin et al., 2013). The grouping of hyperthermophiles at the base of the archaeal tree also suggests that LACA was a hyperthermophile (Brochier-Armanet et al., 2011; Petitjean et al., 2015), whereas LUCA was probably a mesophile or a moderate thermophile (Boussau et al., 2008a; Groussin and Gouy, 2011). Some proposed events are more speculative but supported by theoretical arguments, such as the independent introduction of DNA (blue arrows) from viruses, into the lineages leading to Bacteria and to Archaea/Eukarya (Forterre, 2002) and the thermoreduction (red arrows) at the origin of the modern “akaryotic” phenotype (Forterre, 1995).

Rooting of the domain Eukarya and internal branching in this domain have been adapted from the recent tree of Baldauf and colleagues which is based on a concatenation of mitochondrial proteins rooted with their bacterial homologs (a rather close outgroup compared to Archaea; He et al., 2014). This tree is rooted between Discoba (Jakobida plus Discritata) and all other eukaryotic megagroups. This rooting has been criticized by Derelle et al. (2015) who found distant paralogs in the data set used by He et al. (2014). These authors located the root of the eukaryotic tree between Amorpha and other eukaryotic groups in mitochondrial proteins trees. This rooting corresponds to the previous division between Unikonta and Bikonta originally proposed by (Stechmann and Cavalier-Smith, 2003; Cavalier-Smith, 2010). Derelle et al. (2015) have now suggested naming these two assemblages Optimoda (Amorpha in Figures 3–5) and Diphoda.

The position of the root of the domain Eukarya has been constantly changing with further phylogenetic analyses, raising doubt about the possibility of settling this issue using molecular phylogenetic methods based on protein sequences. I thus decided to root this domain between Jakobida and all other eukaryotes in the universal tree of Figure 3, as recently suggested by Kannan et al. (2014), because Jakobida contain large mitochondrial genomes that still encode the bacterial RNA polymerase genes (Burger et al., 2013; Kamikawa et al., 2014; Kannan et al., 2014). The mitochondria of the LECA probably still had this RNA polymerase, which was subsequently replaced with a viral RNA polymerase in all Eukaryotes, except Jakobida (Kannan et al., 2014). These viral RNA polymerases have been recruited from a provirus integrated into the genome of the alpha-proteobacterium, which gave rise to mitochondria (Filée and Forterre, 2005). It seems unlikely that this non-orthologous replacement occurred twice independently in the history of mitochondria. Accordingly, the rooting between Jakobida and other eukaryotes is reasonable (more parsimonious) because it requires a single NOR of RNA polymerase in mitochondrial evolution, whereas the rooting between Amorpha and other eukaryotes would require several independent non-orthologous replacements.

Rooting of the domain Bacteria and internal branching in this domain have been adapted from the ribosomal protein trees of Koonin and colleagues (Yutin et al., 2012). These authors have suggested several superphyla beside the previously recognized PVC superphylum, which includes Planctomycetes, Verrucomicrobia and Chlamydiae (Kamke et al., 2014). These putative superphyla are indicated by circles in the tree of Figure 3. Branchings within Proteobacteria are drawn according to the ribosomal protein tree of Brochier-Armanet and colleagues (Ramulu et al., 2014). The tree is tentatively rooted between the PVC superphylum and all other Bacteria, according to the basal rooting of Planctomycetes obtained by Brochier and Philippe using slowly evolving positions in ribosomal RNA sequences (Brochier and Philippe, 2002). Bacteroidetes are indicated in the second branching because these Bacteria are grouped with PVC bacteria in the phylogenetic analysis based on ribosomal proteins (Yutin et al., 2012).

The basal position of PVC in the bacterial tree of Figure 3, which remains to be confirmed, is appealing because the ancestor of PVC bacteria contained several genes encoding proteins structurally analogous to various eukaryotic coat proteins involved in vesicle and nuclear pore formation (Santarella-Mellwig et al., 2010). These proteins are probably involved in the invagination of the cytoplasmic membrane that led to the formation of the intracellular cytoplasmic membrane (ICM) in most PVC bacteria. This mimics the role of coat proteins in eukaryotes that are involved in the formation of the endoplasmic reticulum and nuclear membranes. The basal position of PVC bacteria suggests a parsimonious scenario in which these proteins were present in LUCA, and later on lost in Archaea and most Bacteria (Forterre and Gribaldo, 2010). However, this scenario is still controversial since it is presently unclear whether the structurally analogous proteins of PVC Bacteria and Eukarya are also homologous (McInerney et al., 2011; Devos, 2012). These proteins are formed by the fusion of two domains (one rich in alpha helices, the other in beta strands) that are each present in the three domains. Accordingly, they can also have originated independently by the fusion of these domains in the branches leading to Eukarya and PVC bacteria.

Archaea have been tentatively rooted in the branch leading to Lokiarchaeota in the tree of Figure 3, because this candidate phylum contains most eukaryotic features present in Archaea and branches closer to Eukarya than to other archaea in phylogenetic analyses of universal proteins, even when bacterial proteins are removed from the analysis (Spang et al., 2015, Figure S13D). Previously, the archaeal ribosomal tree was rooted in the branch leading to Thaumarchaeota when eukaryotic proteins were used as outgroup (Brochier-Armanet et al., 2008a). This rooting was also observed in a phylogenetic analysis of the archaeal replicative helicase MCM, which is a good phylogenetic marker for the archaeal domain (Krupovic et al., 2010), and in a phylogeny of five informational proteins present in deeply branching Thaumarchaeota from Kamchatkan thermal springs (Eme et al., 2013). I thus place Thaumarchaeota as the second branch in the archaeal subtree.

Moreira and colleagues, using bacterial proteins (including ribosomal proteins) as outgroup, have recently proposed to root the archaeal tree in the branch leading to Euryarchaeota (Petitjean et al., 2014). As a consequence, they propose to create a new phylum, Proteoarchaeota, grouping Crenarchaeota and Thaumarchaeota, together with the putative phyla Aigarchaeota and Korarchaeota. Proteoarchaea thus corresponds to the previously so-called “TACK superphylum” (Thaumarchaeota, “Aigarchaeota,” Crenarchaeota, Korarchaeota). However, as previously mentioned, using the same strategy, Gribaldo and colleagues obtained a root located within Euryarchaeota, more precisely in between subphyla I and II (Raymann et al., 2015). In contrast, their archaeal tree is rooted between “TACK/Proteoarchaeota” and Euryarchaeota when they used eukaryotic proteins as an outgroup. It will be interesting to see if they recover the root in the branch leading to Lokiarchaeota in future analyses using eukaryotic sequences as an outgroup.

Moreira and colleagues argue that eukaryotic proteins cannot be used to root the archaeal tree if Eukarya emerged from within Archaea. However, in the framework of the classical Woese tree, it makes more sense to root the archaeal tree using eukaryotic proteins as outgroup, because these proteins are much more closely related than bacterial proteins to their archaeal orthologs. Notably, the rooting between Lokiarchaeota/Thaumarchaeota and other Archaea, obtained in that case is more parsimonious than the rooting between Euryarchaeota and other Archaea in explaining the presence in Lokiarchaeota/Thaumarchaeota (including “Aigarchaeota,” see below) of many eukaryotic features lacking in other Archaea (Brochier-Armanet et al., 2008b; Spang et al., 2010, 2015; Koonin and Yutin, 2014).

Euryarchaeota are divided in two sub-phyla I and II, according to the presence/absence of DNA gyrase, a bacterial DNA topoisomerase that was transferred once in the phylum Euryarchaeota (Raymann et al., 2014). The sub-phylum I Euryarchaeota corresponds to those lacking DNA gyrase and encompasses Thermococcales*, Nanoarchaeum*, and class I methanogens, whereas sub-phylum I corresponds to those containing DNA gyrase and encompasses Archaeoglobales, Thermoplasmatales, Halobacteriales, and class II methanogens (Forterre et al., 2014b).

Phylogenetic analyses have shown that DNA gyrase has been transferred from Bacteria to Archaea (Raymann et al., 2014). This transfer was an important and unique event that had a critical impact on chromosome structure and patterns of gene expression. Indeed, plasmids from all archaea are relaxed or slightly positively supercoiled, whereas plasmids from member of sub-phylum II Euryarchaeota containing gyrase are negatively supercoiled (Forterre et al., 2014b). Once transferred, DNA gyrase became most likely essential, as demonstrated in the case of Halobacteriales and Methanococcales, because such drastic modification in DNA topology modifies all protein DNA interactions involved in replication and transcription (for review and discussion, see Forterre and Gadelle, 2009). Indeed, to date, the loss of DNA gyrase has not been reported in any organism. Importantly, the phylogeny of archaeal DNA gyrase is fully congruent with the phylogeny of sub-phylum II Euryarchaeota, suggesting that, once transferred to the ancestor of this group, DNA gyrase has co-evolved with sub-phylum II Euryarchaeota (Raymann et al., 2014). Accordingly, considering the importance of DNA gyrase in cell physiology (DNA topology controlling all gene expression patterns) I suggest calling sub-phylum II Euryarchaeota, the neo-euryarchaeota. This name emphasizes the fact that the ancestor of this sub-phylum lived after the formation of the major bacterial phyla, since archaeal DNA gyrases branch within bacterial ones (Raymann et al., 2014).

Since all rooting indicated in the tree of Figure 3, as well as most internal nodes within domains, are controversial, I present a second tree (Figure 4), in which the information is limited to only that accepted by consensus. Accordingly, each one of the three domains is shown in a radial form without roots and only a few nodes within domains that seem supported by strong phylogenetic analyses are indicated (Brochier-Armanet et al., 2008a; He et al., 2014; Kamke et al., 2014; Ramulu et al., 2014; Spang et al., 2015).

Finally, I also present a third tree in which Eukarya emerged from within Archaea (Figure 5). This tree includes the new root proposed by Gribaldo and co-workers for Archaea (Raymann et al., 2015) and shows Lokiarchaeota as sister group of Eukarya (Spang et al., 2015). Notably, if future analyses demonstrate that such a tree is the more likely tree, Archaea will not be a valid taxon anymore, except if one accepts to consider eukaryotes as a particular archaeal phylum (much like *Homo* is a particular lineage of Apes)! In that case, the name Arkarya could be substituted to Archaea. Eukarya will become a particular phylum of Arkarya, beside Euryarkaryota, Crenarkaryota, Thaumarkaryota, and Lokiarkaryota (Figure 5).

As can be seen, Figures 4 and 5 can be easily deduced from Figure 3. This indicates that it will be easy to update and modify these trees following the accumulation of new data from comparative genomics and phylogenetic analyses.

## The Archaeal Tree

**FIGURE 6:**
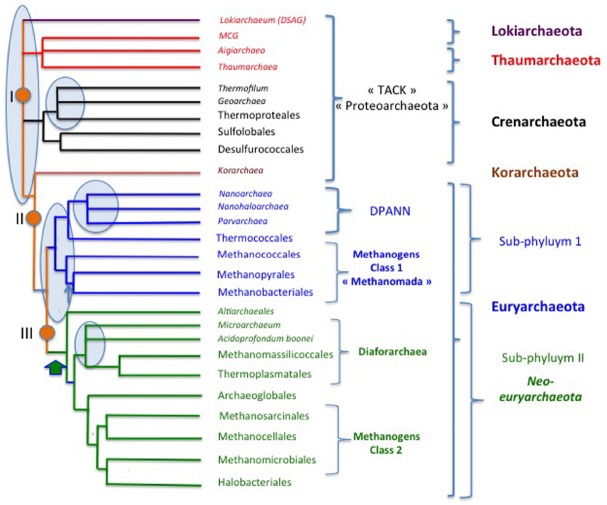
**Schematic tree of the archaeal domain.** The alternative proposed roots are from : I (Brochier-Armanet et al., 2008a), II (Petitjean et al., 2014), III (Raymann et al., 2015). Thin blue arrow: emergence of pseudomurein; large green arrow, introduction of DNA gyrase from Bacteria (Raymann et al., 2014), pale blue ovals emphasize poorly resolved, controversial nodes.

Figure 6 illustrates a rather detailed, but schematic, archaeal tree as a tribute to this issue devoted to Archaea. This tree has been adapted from the ribosomal protein tree of Brochier-Armanet et al. (2011) and from a recent phylogeny based on the concatenation of 273 proteins conserved in at least 119 archaeal species out of 129 (Petitjean et al., 2015; thereafter called the archaeal protein tree). I also include the recently described candidate phylum “Lokiarchaeota” (corresponding to the DSAG clade) considering its importance for the discussions about the origin of Eukarya. The various roots that have been proposed for the domain Archaea are indicated by orange circles.

Aigarchaeota are included within Thaumarchaeota, because the latter were originally defined as a major archaeal phylum encompassing all archaeal lineages that are sister groups of Crenarchaeota in rDNA analyses (Brochier-Armanet et al., 2011). In the original paper in which we propose this new phylum, we noticed that: “*The diversity of mesophilic crenarchaeota—*that we proposed to rename Thaumarchaeota*—based on SSU rRNA sequence is comparable to that of hyperthermophilic Crenarchaeota and Euryarchaeota, which suggests that they represent a major lineage that has equal status to Euryarchaeota and Crenarchaeota*” (Brochier-Armanet et al., 2008a). We also predicted that the mesophily of Thaumarchaeota “*could be challenged by the future identification of non-mesophilic organisms that belong to this phylum*.” This suggests considering *candidatus* Caldarchaeum subterraneum as a thermophilic member of the Thaumarchaeota and not as the prototype of a new phylum (Aigarchaeota). In agreement with this proposal, *candidatus* C. subterraneum emerges as sister group of other thaumarchaea in most phylogenies (Brochier-Armanet et al., 2011; Nunoura et al., 2011; Eme et al., 2013; Guy et al., 2014; Petitjean et al., 2014, 2015; Raymann et al., 2015; Spang et al., 2015). Furthermore, *candidatus* C. subterraneum exhibits all molecular features first used to define the phylum Thaumarchaeota (Brochier-Armanet et al., 2008a; Spang et al., 2010), such as a eukaryotic-like Topo IB, which is absent from all other Archaea (Brochier-Armanet et al., 2008b). Topo IB is absent in Lokiarchaeota, but one must bear in mind that the reconstituted genome is only 92% complete (Spang et al., 2015). The phylum Thaumarchaeota should also include uncultivated archaea from the clade MCG (the Miscellaneous Crenarchaeal Group), since these organisms systematically form monophyletic groups with Thaumarchaea and “Aigarchaeota” in phylogenetic analyses (Spang et al., 2015).

The recently proposed phylum “Geoarchaeota” is included as a sister group of Thermoproteales (without phylum status) as suggested by the analysis of Ettema and co-workers (Guy et al., 2014; Spang et al., 2015). *Thermofilum* always branch very early as a sister group of Thermoproteales. This suggests that *Thermofilum*, as well as Geoarchaeota, could have an order status.

Korarchaeota branch in-between Euryarchaeota and other archaea (Crenarchaeota, Thaumarchaeota) in the ribosomal proteins tree. Unfortunately, this phylum is presently represented by a single species whose genome has been sequenced, *candidatus* Korarchaeum cryptofilum. The genome of *candidatus* K. cryptofilum harbors a mixture of features characteristic of the three other archaeal phyla. This can justify maintaining a phylum status for this group for the moment. More genome sequences of Korarchaeota are nevertheless required to confirm this point.

As previously discussed, Euryarchaeota are divided into two groups depending of the presence of DNA gyrase. Neo-euryarchaeota (sub-phylum II) is a monophyletic group in all phylogenetic analyses. In contrast, sub-phylum I is paraphyletic in the ribosomal and archaeal protein trees (Brochier-Armanet et al., 2011; Petitjean et al., 2015). However, they form a monophyletic assemblage in a tree based on replication proteins (Raymann et al., 2014) and in a recent phylogenomic analysis performed by Makarova et al. (2015) that involved both comparison of multiple phylogenetic trees and a search for putative synapomorphies. I thus decided to favor the monophyly of sub-phylum I in the tree of Figure 6.

The close relationship between plasmids of Thermococcales and Methanococcales also suggests that these two orders could be closely related (Soler et al., 2010). It is possible that the emergence of pseudomurein in Methanobacteriales and Methanopyrales allowed these archaea to get rid of mobile elements that used to infect the ancestors of Thermococcales and Methanococcales. Notably, Methanopyrales and Methanobacteriales are monophyletic in the archaeal protein tree, suggesting that the presence of pseudomurein is a synapomorphy for these sister groups (Petitjean et al., 2015). Petitjean and coworkers recently proposed the name Methanomada (superclass) for class I methanogens, that are monophyletic in their protein tree (Petitjean et al., 2015) and in the DNA replication tree (Raymann et al., 2014).

In contrast to class I, the four orders of class II methanogens that are included within neo-euryarchaeota are always paraphyletic in phylogenetic analyses. Methanogens of the recently described order Methanomassiliicoccus form a monophyletic assemblage with Thermoplasmatales, the moderate thermoacidophilic strain *Aciduliprofundum boonei* and several lineages of uncultivated archaea in a ribosomal protein tree (Borrel et al., 2013). The name Diafoarchaea has been proposed for this major subgroup (superclass) of neo-Euryarchaeota (Petitjean et al., 2015).

Altiarchaeales correspond to a recently described mesophilic archaeum, *Candidatus* Altiarchaeum hamiconexum, characterized by fascinating appendages (Hami) that groups with Methanococcales in a ribosomal protein tree, but between Euryarchaeota of sub-phyla I and II in a tree based on several other universal proteins (Probst et al., 2014). Since *Candidatus* A. hamiconexum contain the two DNA gyrase genes, it is located at the base of the neo-euryarchaeota in the tree of Figure 6.

Rinke et al. (2013) have recently proposed promoting the nanosized archaea *Nanoarchaeum*, *Parvarchaeum* (ARMAN 4 and 5) and Nanohaloarchaea to phylum level and grouping them with two other putative new phyla of Archaea (“Aenigmarchaeota” and “Diapherotrites”) into a new superphylum, called DPANN (Rinke et al., 2013). In their phylogenetic analysis based on 38 “universal proteins,” the root of the archaeal tree is located between this putative DPANN superphylum and all other Archaea (see Figure S11 in Rinke et al., 2013). However, their universal protein data set is confusing because it contains eukaryotic proteins of bacterial origin (Williams and Embley, 2014). In the recent tree of Williams and Embley supporting the archaeal origin of eukaryotes, the archaeal tree is rooted between DPANN and Euryarchaeota (see Figure 3 in Williams and Embley, 2014). The basal position of the nanosized archaea in these trees confirms the difficulty of using universal proteins to resolve ancient phylogenies, because this position is clearly misleading (see below and Petitjean et al., 2014). In the case of nanosized archaea, phylogenetic analyses are even more challenging because their proteins tend to evolve rapidly, producing very long branches in phylogenetic trees. This suggests that these nanosized archaea evolve mainly by streamlining (Brochier-Armanet et al., 2011; Petitjean et al., 2014). They have not been included in the protein tree of Petitjean and coworkers (Petitjean et al., 2015).

Previous phylogenetic and comparative genomic analyses focusing on *Nanoarchaeum equitans* have suggested that this fascinating archaeal symbiont belongs to the Euryarchaeota and could be distant relatives of Thermococcales (Brochier et al., 2005b). This sister relationship was later on supported by the discovery of the shared presence of a tRNA modification protein that was recently transferred from Bacteria to both *N. equitans* and Thermococcales (Urbonavicius et al., 2008). The sisterhood of *N. equitans* and Thermococcales has been observed again in more recent analyses based on ribosomal proteins (Brochier-Armanet et al., 2011) or in the archaeal tree of Moreira and colleagues (Petitjean et al., 2014).

In the tree of Figure 6, Thermococcales, *Nanoarchaeum*, *Parvarchaeum*, and Nanohaloarchaea tentatively form a monophyletic group. This is supported by several lines of converging (but weak and controversial) evidences. The grouping of Thermococcales and *Nanoarchaeum* with *Parvarchaeum* (ARMAN 4 and 5) is supported by the phylogeny based on ribosomal proteins (Brochier-Armanet et al., 2011), whereas the grouping of *Nanoarchaeum* and *Parvarchaeum* with Nanohaloarchaea is supported by the phylogeny of DNA replication proteins (Raymann et al., 2014). Interestingly, the grouping of *Parvarchaeum*, *Nanoarchaeum*, and Nanohaloarchaea is supported by the shared presence of an atypical small primase corresponding to the fusion of the two subunits of the *bona fide* archaeal/eukaryal primases, PriS, and PriL (Raymann et al., 2014). It has been suggested that this fusion corresponds to a convergent evolution associated to streamlining (Petitjean et al., 2014). However, this is unlikely because these unusual monomeric primase primases are also highly divergent in terms of amino-acid sequence from the classical archaeal/eukaryal primases and very similar in all nanosized archaea. Alternatively, it has been suggested that this primase has been distributed between nanosized archaea by horizontal gene transfers (Raymann et al., 2014). This seems also unlikely because nanosized archaea live in very different types of environments (high temperature for known *Nanoarchaeum*, high salt for Nanohaloarchaea, high acidity for *Parvarchaeum*). It seems more likely that this primase was acquired by a common ancestor to *Nanoarchaeum*, *Parvarchaeum*, and Nanohaloarchaea from a mobile genetic element (Raymann et al., 2014). It has indeed been shown that some archaeal plasmids encode unusual primases from the PriS/PriL superfamily that are only distantly related to *bona fide* archaeal and eucaryal primases (Lipps et al., 2004; Krupovic et al., 2013; Gill et al., 2014).

The grouping of Nanohaloarchaea with other nanosized archaea, as in Figure 6, is especially controversial because they emerge as sister group of Halobacteriales in a ribosomal protein tree (Narasingarao et al., 2012). However, if Nanohaloarchaea are sister group of Halobacteriales, they would be the only members of neo-euryarchaeota lacking DNA gyrase. Nanohaloarchaea might have lost this enzyme during the streamlining process related to their small size. However, the loss of DNA gyrase has not been reported until now in any other free-living organisms. This is why I finally choose to group Nanohaloarchaea with other nanosized archaea in the tree of Figure 6. It is possible that the atypical amino acid composition of the nanohaloarchaeal proteome (Narasingarao et al., 2012), linked to salt adaptation, introduces a bias favoring the artificial grouping of Nanohaloarchaea and Haloarchaea. This would also explain why Nanohaloarchaea attracts *Nanoarchaeum* and *Parvarchaeum* away from Thermococcales and closer to Haloarchaea in the DNA replication tree (Raymann et al., 2014).

The nanosized archaeon *Candidatu*s Micrarchaeum acidophilum (ARMAN 2) branches together with *Parvarchaeum* (ARMAN 4, 5) in a rDNA tree (Baker et al., 2006) and in the tree of Moreira and coworkers (Petitjean et al., 2014). However, it branches away from *Parvarchaeum* in the ribosomal tree, as an early branching neo-euryarchaeon. The latter position is supported by the presence of DNA gyrase in *Candidatus* Micrarchaeum acidophilum and the absence of the single subunit primase characteristic of other nanosized archaea (Raymann et al., 2014). I thus included *Micrarchaeum* among the superclass Diafoarchaea in the tree of Figure 6. Finally, the phylogenetic position and status of “Aenigmarchaeota” and “Diapherotrites” cannot presently be determined because these groups have only been defined from single cell genomic analyses and their genomes are probably incomplete (Petitjean et al., 2014).

Further analyses and (hopefully) many more isolates are clearly required to determine the correct position of the various groups of nanosized archaea in the archaeal tree. From all these considerations, it is clear that the “DPNN superphylum” is an artificial construction. The same is true for the “TACK superphylum” and the candidate phylum “Proteoarchaea” if the root of the archaeal tree is located within Euryarchaeota (Raymann et al., 2015) or between Thaumarchaeota/Lokiarchaeota and other Archaea. As we previously discussed, defining the root of the archaeal tree strongly depends on choosing between different scenarios for the universal tree. Since the rooting of the archaeal tree is still in debate, I would not recommend at the moment to use names such as “Proteoarchaea” or “TACK superphylum” in archaeal phylogeny.

This section on archaeal phylogeny has illustrated the fact that, in addition to the root of the tree itself, several nodes in the archaeal tree are still controversial and require more data and more work to be carried out. These nodes have been marked by circles in blue in the tree of Figure 6. Future progress will probably come from the sequencing of more genomes, especially in poorly represented groups and in the many groups that are presently only known from environmental rDNA sequences.

## Conclusion

I hope that the universal and archaeal trees proposed here will be useful as new metaphors illustrating the history of life on our planet. From the above review, it should be clear that there is no protein or groups of proteins that can give the real species tree, i.e., allow us to recapitulate safely the exact path of life evolution. In particular, one should be cautious with composite trees based on the concatenation of protein sequences or addition of individual trees. The results obtained should always be compared to the result of individual trees. Martin and colleagues recently reported a lack of correspondence between individual protein trees and the concatenation tree in several datasets of archaeal and bacterial proteins (Thiergart et al., 2014). This can reflect either LGT and/or the absence of real phylogenetic signal. Importantly, this lack of correspondence between individual protein trees and concatenation trees is also observed in the analyses that place Eukarya within Archaea (Cox et al., 2008; Williams and Embley, 2014), raising doubts on results supporting the tree of Figure 5. Unfortunately, individual trees are not always available in the supplementary data of published studies (Katz and Grant, 2014; Raymann et al., 2015). In our previous work on archaeal phylogeny, careful analysis of individual trees in parallel to their concatenation was critical to obtain a rather confident tree for Archaea based on ribosomal proteins (Matte-Tailliez et al., 2002) and to find the (probably) correct position of Nanoarchaeota, as sister group of Thermococcales (Brochier et al., 2005b). Notably, to obtain this result in Brochier et al. (2005a), it was necessary to remove the proteins from the large ribosome subunit because several of them exhibit a surprising affinity with their crenarchaeal homologs in individual trees, possibly indicating LGT between *N. equitans* and its host, the crenarchaeon *Ignicoccus*. It is also very important to compare trees obtained with different datasets, such as translation, transcription and DNA replication trees, to pint-point discrepancies and identify their causes (Brochier et al., 2004, 2005a; Raymann et al., 2014).

In summary, it is (and it will be) only possible to draw schematic (theoretical) trees by combining data from multiple phylogenetic protein trees with information deduced from probable synapomorphies. It is the exercise that I have tried to do here in drawing the trees of Figures 3–6. These organismal trees, which are supposed to recapitulate the history of ribosome encoding organisms, will probably evolve themselves, with the availability of new genomes (especially from poorly sampled groups), better phylogenetic analyses, and the identification of new synapomorphies defining specific domains, sub-phyla groups and superphyla. For instance, my preliminary analyses of universal proteins sequence alignments indicate that the Lokiarchaeon is probably neither an early braching archaeon nor a missing link between Archaea and Eukarya (see also Nasir et al., 2015). It should be relatively easy to update these trees as new data accumulate in the future and use them in discussions of various controversial scenarios regarding the evolution of ancient life.

### Conflict of Interest Statement

The author declares that the research was conducted in the absence of any commercial or financial relationships that could be construed as a potential conflict of interest.
